# Supplementary material for: Selective Inhibition of Soluble Tumor Necrosis Factor Alters the Neuroinflammatory Response following Moderate Spinal Cord Injury in Mice
Source: Biology (Basel). 2023 Jun 12;12(6):845. doi: 10.3390/biology12060845 (PMC10295415; doi:10.3390/biology12060845)
Supplement: Supplementary file 1 [file biology-12-00845-s001.zip › biology-2315353-supplementary.pdf]

biology

MDPI

Supplemental Material

Selective inhibition of soluble tumor necrosis factor alters the neuroinflammatory response following moderate spinal cord injury in mice

Minna Christiansen Lund [1,§], Ditte Gry Ellman [1,§], Pernille Vinther Nielsen[1,2], Stefano Raffaele[1,3], Marta Fumagalli[3], Raphael Guzman[4], Matilda Degn[5], Roberta Brambilla[1,6,7,8], Morten Meyer[1,2,7], Bettina Hjelm Clausen[1,7] and Kate Lykke Lambertsen [1,2,7,,*]

1 Department of Neurobiology Research, Institute of Molecular Medicine, University of Southern Denmark, Odense, Denmark; minnacll@hotmail.com; dellman@health.sdu.dk; pvnielsen@health.sdu.dk; stefano.raffaele@unimi.it; rbrambilla@med.miami.edu; mmeyer@health.sdu.dk; bclausen@health.sdu.dk; klambertsen@health.sdu.dk

2 Department of Neurology, Odense University Hospital, Odense, Denmark

3 Department of Pharmacological and Biomolecular Sciences, Università degli Studi di Milano, Milan, Italy; marta.fumagalli@unimi.it

4 Department of Biomedicine, University of Basel, Basel, Switzerland; raphael.guzman@usb.ch

5 Department of Paediatrics and Adolescent Medicine, Rigshospitalet, Copenhagen, Denmark; matildadegn@hotmail.com

6 The Miami Project to Cure Paralysis, Department of Neurological Surgery, University of Miami Miller School of Medicine, Miami, FL, USA

7 Brain Research Inter-Disciplinary Guided Excellence (BRIDGE), Department of Clinical Research, Odense, Denmark

8 Neuroscience Program, University of Miami Mille School of Medicine, Miami, FL 33136, USA

§ Shared first authors

* Correspondence: klambertsen@health.sdu.dk; Tel.: +45 6550 3806

Table S1. Urine content and weight for SCI mice treated with saline or XPro1595.

	Saline	XPro1595	Multiple t-test, p-value
Urine content (g)			
1d after SCI	0.43 ± 0.03	0.42 ± 0.04	t_{48}=0.16, p=0.87
3d after SCI	0.31 ± 0.03	0.37 ± 0.04	t_{48}=1.08, p=0.29
7d after SCI	0.23 ± 0.05	0.30 ± 0.06	t_{48}=0.84, p=0.40
14d after SCI	0.18 ± 0.04	0.14 ± 0.05	t_{48}=0.53, p=0.60
21d after SCI	0.14 ± 0.05	0.14 ± 0.05	t_{48}=0.00, p>0.99
28d after SCI	0.15 ± 0.07	0.08 ± 0.06	t_{22}=0.74, p=0.47
35d after SCI	0.21 ± 0.10	0.20 ± 0.10	t_{22}=0.06, p=0.95
Weight (g)			
Baseline	21.08 ± 0.37	20.92 ± 0.37	t_{48}=0.29, p=0.77
1d after SCI	19.48 ± 0.34	19.84 ± 0.34	t_{48}=0.76, p=0.46
3d after SCI	18.41 ± 0.37	18.69 ± 0.36	t_{48}=0.55, p=0.58
7d after SCI	19.88 ± 0.34	20.12 ± 0.38	t_{48}=0.48, p=0.64
14d after SCI	21.10 ± 0.33	21.67 ± 0.41	t_{48}=1.08, p=0.29
21d after SCI	21.47 ± 0.34	21.98 ± 0.36	t_{48}=1.02, p=0.31
28d after SCI	22.32 ± 0.49	22.86 ± 0.36	t_{22}=0.89, p=0.39
35d after SCI	22.38 ± 0.59	23.01 ± 0.46	t_{22}=0.83, p=0.41

Multiple unpaired t-test. Days 1-21: n=25/treatment group; Days 28-35: n=12/treatment group.

Table S2. Cytokine and TNF receptor protein levels in sham mice.

	1 hour after sham surgery		1 day after sham surgery	
	**Saline**	**XPro1595**	**Saline**	**XPro1595**
**TNF (pg/mg)**	0.94 ± 0.14	0.94 ± 0.26	0.70 ± 0.14	1.0 ± 0.20
**TNFR1 (pg/mg)**	126.83 ± 34.07	118.80 ± 20.35	237.81 ± 68.70	270.92 ± 66.72
**TNFR2 (pg/mg)**	90.83 ± 38.18	79.77 ± 19.95	139.82 ± 57.60	161.58 ± 48.84
**IL-1β (pg/mg)**	0.11 ± 0.03	0.14 ± 0.07	1.39 ± 0.89	1.84 ± 1.08
**IL-6 (pg/mg)**	10.87 ± 6.48	11.88 ± 6.35	146.97 ± 144.18	151.23 ± 61.20
**IL-10 (pg/mg)**	0.27 ± 0.18	0.21 ± 0.07	0.24 ± 0.14	0.22 ± 0.15
**CXCL1 (pg/mg)**	1.55 ± 0.25	1.73 ± 0.92	30.77 ± 14.93	23.10 ± 7.74

TNF levels (Time: $F_{1,16}$=0.22, p=0.64, Treatment: $F_{1,16}$=0.64, p=0.43, Interaction: $F_{1,16}$=0.60, p=0.45), TNFR1 (Time: $F_{1,16}$=32.21, p<0.0001, Treatment: $F_{1,16}$=0.29, p=0.60, Interaction: $F_{1,16}$=0.79, p=0.39), TNFR2 (Time: $F_{1,16}$=11.32, p<0.004. Treatment: $F_{1.16}$=0.08. p=0.79. Interaction: $F_{1.16}$=0.71. p=0.41), IL-1β (Time: $F_{1,14}$=17.56, p<0.0009, Treatment: $F_{1,14}$=0.47, p=0.50, Interaction: F_{114}=0.35, p=0.56), IL-6 (Time: $F_{1,16}$=15.41, p<0.001, Treatment: $F_{1,16}$=0.006, p=0.94, Interaction: $F_{1,16}$=0.002, p=0.96), IL-10 (Time: $F_{1,15}$=0.01, p=0.90, Treatment: $F_{1,15}$=0.40, p=0.54, Interaction: $F_{1,15}$=0,16, p=0,70), and CXCL1 (Time: $F_{1,16}$=29,03, p<0,0001, Treatment: $F_{1,16}$=0,11, p=0,74, Interaction: $F_{1,16}$=0,08, p=0,78) levels were comparable between treatment groups. Two-way ANOVA with Sidak's multiple comparison test. Data are presented as mean±SEM with n=5/treatment group/time point.

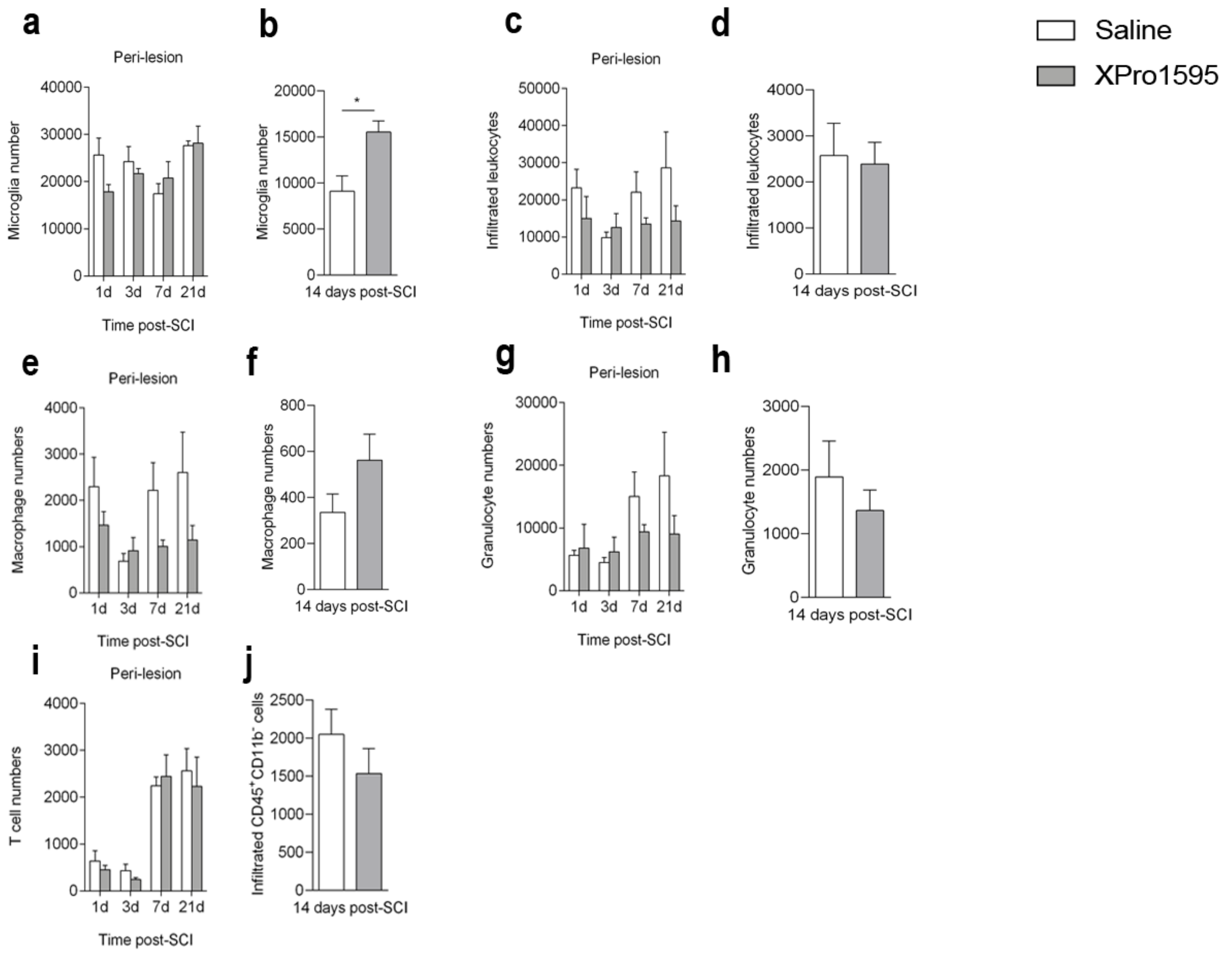

Figure S1. Flow cytometric analysis of peri-lesion areas after SCI. (**a-b**) Total number of microglia ($CD11b^+CD45^{dim}$) at day 1, 3, 7, and 21 after SCI (a, Interaction: $F_{3,32}$=1.58, p=0.21; Time: $F_{3,32}$=3.79, p=0.02; Treatment: $F_{1,32}$=0.74, p=0.40) and at day 14 (b, t_8=3.11, p=0.01). (**c-d**) Total number infiltrating leukocytes ($CD11b^+CD45^{high}$) at day 1, 3, 7, and 21 after SCI (c, Interaction: $F_{3,32}$=0.94, p=0.043; Time: $F_{3,32}$=1.43, p=0.25; Treatment: $F_{2,32}$=3.69, p=0.06) and at day 14 (d, t_8=0.22, p=0.83). (**e-f**) Total number of macrophages ($Ly6C^+Ly6G^-$) at day 1, 3, 7, and 21 after SCI (g, Interaction: $F_{3,32}$=1.20, p=0.32; Time: $F_{3,32}$=2.26, p=0.10; Treatment: $F_{1,32}$=5.86, p=0.02) and at day 14 (h, t_8=1.64, p=0.14). (**g-h**) Total number of granulocytes ($Ly6C^+Ly6G^+$) at day 1, 3, 7, and 21 after SCI (g, Interaction: $F_{3,32}$=1.20, p=0.33; Time: $F_{3,32}$=2.97, p=0.05; Treatment: $F_{1,32}$=1.52, p=0.23) and at day 14 (h, t_8=0.82, p=0.44). (**i**) Total number of T-cells at day 1, 3, 7, and 21 after SCI (Interaction: $F_{3,32}$=0.22, p=0.88; Time: $F_{3,32}$=21.30, p<0.0001; Treatment: $F_{1,32}$=0.27, p=0.61). (**j**) Total numbers of lymphocytes ($CD45^+CD11b^-$) at day 14 after SCI (t_8=1.11, p=0.30). Two-way ANOVA and multiple unpaired t-test with n=5/treatment group/time point. Data are presented as mean±SEM. *p<0.05

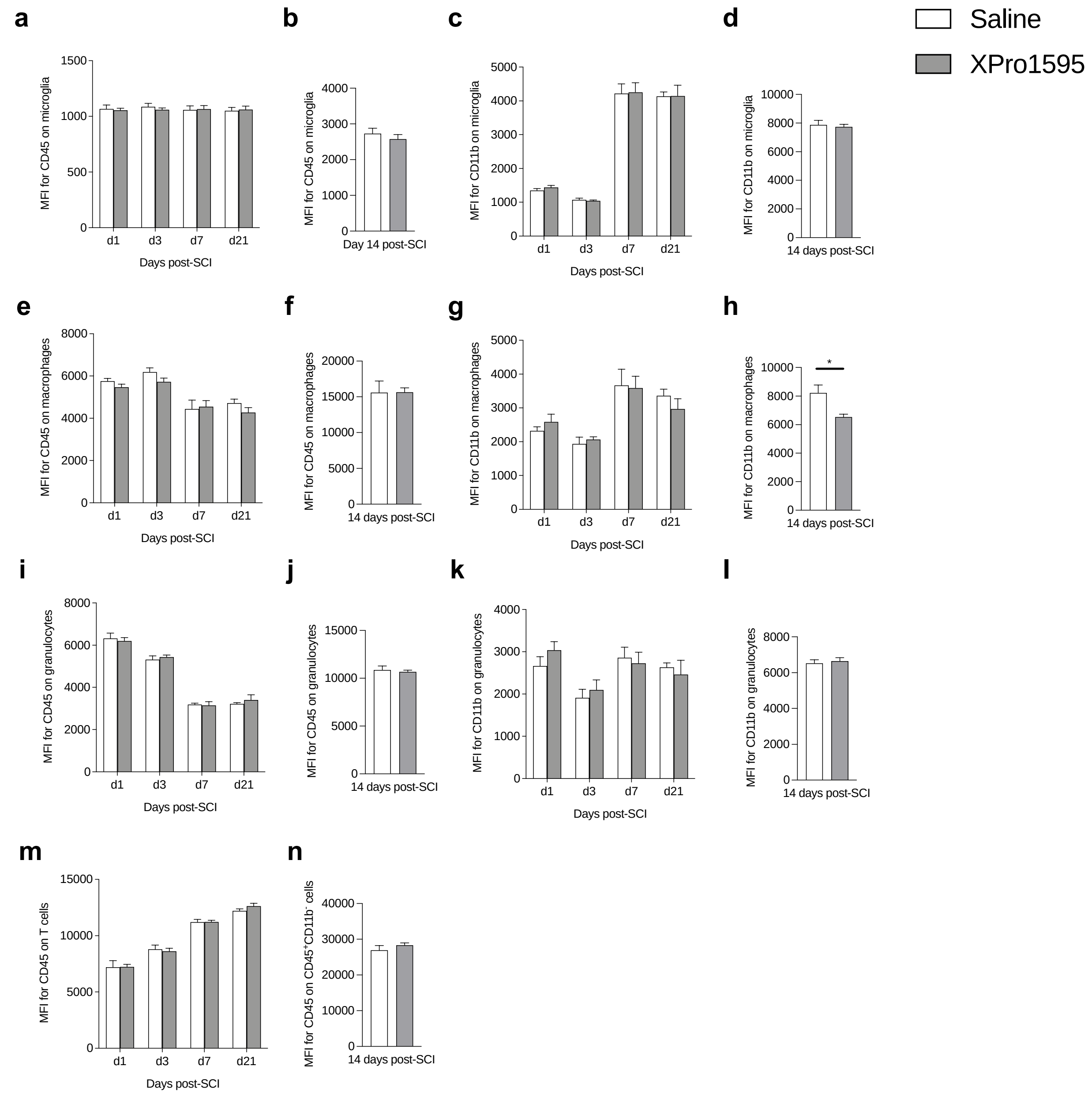

Figure S2. MFI for CD45 and CD11b in the lesion area after SCI. (**a,b**) MFI for CD45 on microglia 1, 3, 7, and 21 days (a, Interaction: $F_{3,32}=0.16$, p=0.92; Time: $F_{3,32}=0.12$, p=0.95; Treatment: $F_{1,32}=0.07$, p=0.79) and 14 days (b, $t_8=0.8$, p=0.45) after SCI. (**c,d**) MFI for CD11b on microglia 1, 3, 7, and 21 days (c, Interaction: $F_{3,32}=0.04$, p=0.99; Time: $F_{3,32}=166.9$, p<0.0001; Treatment: $F_{1,32}=0.04$, p=0.85) and 14 days (d, $t_8=0.4$, p=0.7) after SCI. (**e,f**) MFI for CD45 on macrophages 1, 3, 7, and 21 days (e, Interaction: $F_{3,32}=0.63$, p=0.6; Time: $F_{3,32}=20.49$, p<0.0001; Treatment: $F_{1,32}=2.54$, p=0.12) and 14 days (f, $t_8=0.03$, p=0.98) after SCI. (**g,h**) MFI for CD11b on macrophages 1, 3, 7, and 21 days (g, Interaction: $F_{3,32}=0.54$, p=0.66; Time: $F_{3,32}=14.25$, p<0.0001; Treatment: $F_{1,32}=0.01$, p=0.92) and 14 days (h, $t_8=2.92$, p=0.02) after SCI. (**i,j**) MFI for CD45 on granulocytes 1, 3, 7, and 21 days (i, Interaction: $F_{3,32}=0.33$, p=0.81; Time: $F_{3,32}=166.8$, p<0.0001; Treatment: $F_{1,32}=0.1$, p=0.75) and 14 days (j, $t_8=0.41$, p=0.7) after SCI. (**k,l**) MFI for CD11b on granulocytes 1, 3, 7, and 21 days (k, Interaction: $F_{3,32}=0.61$, p=0.61; Time: $F_{3,32}=5.42$, p=0.004; Treatment: $F_{1,32}=0.15$, p=0.70) and 14 days (l, $t_8=0.43$, p=0.7) after SCI. (**m**) MFI for CD45 on T cells 1, 3, 7, and 21 days after SCI (Interaction: $F_{3,32}=0.34$, p=0.8; Time: $F_{3,32}=117.0$, p<0.0001; Treatment: $F_{1,32}=0.12$, p=0.73). (**n**) MFI for CD45 on $CD45^+CD11b^-$ lymphocytes 14 days after SCI (b, $t_8=0.94$, p=0.37). Two-way ANOVA and multiple unpaired t-test with n=5/treatment group/time point. Data are presented as mean±SEM. *p<0.05.

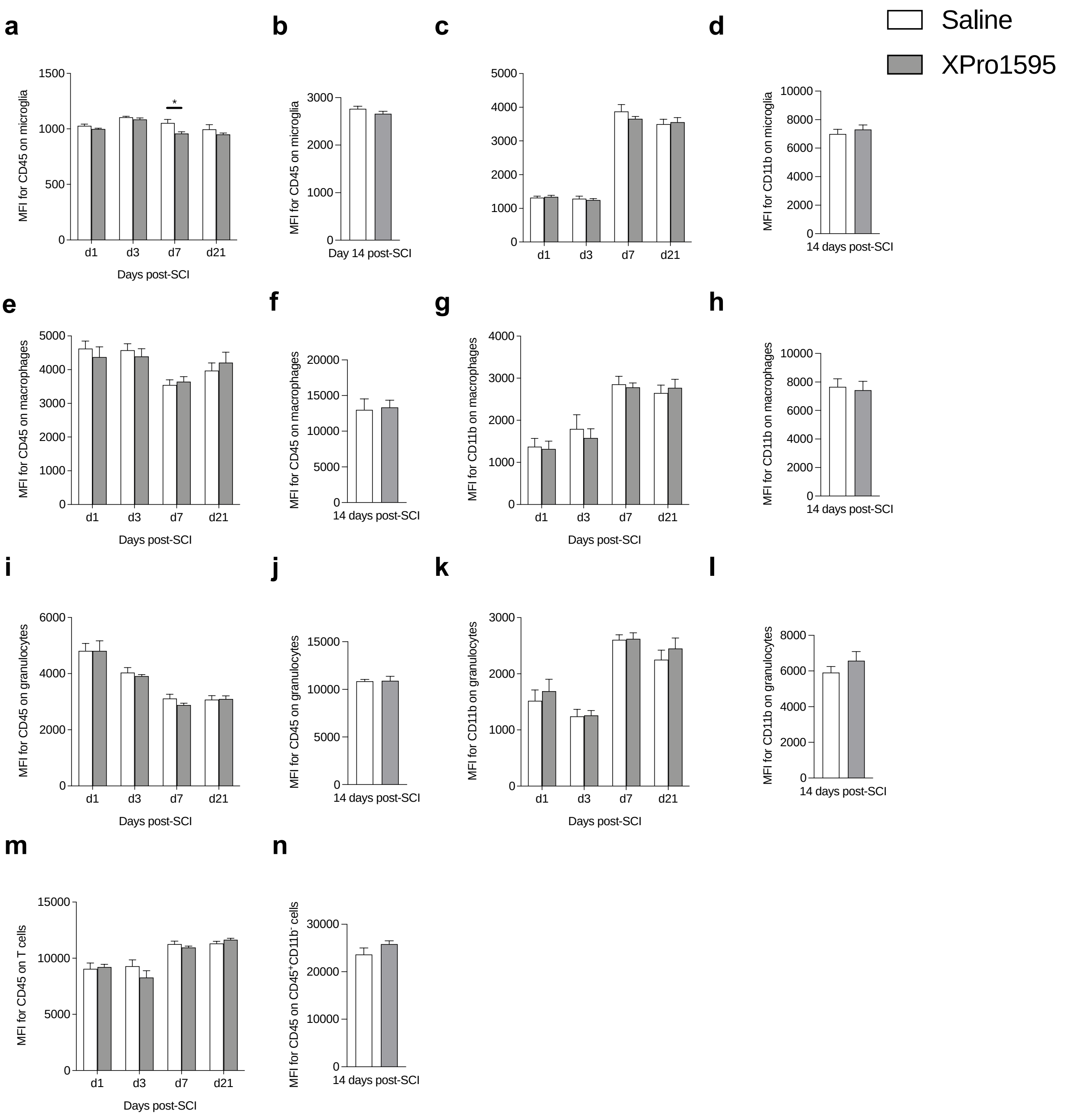

Figure S3. MFI for CD45 and CD11b in the peri-lesion area after SCI. (**a**,**b**) MFI for CD45 on microglia 1, 3, 7, and 21 days (a, Interaction: $F_{3,32}=1.27$, p=0.30; Time: $F_{3,32}=11.7$, p<0.0001; Treatment: $F_{1,32}=10.08$, p=0.003) and 14 days (b, $t_8=1.41$, p=0.19) after SCI. (**c**,**d**) MFI for CD11b on microglia 1, 3, 7, and 21 days (c, Interaction: $F_{3,32}=0.66$, p=0.58; Time: $F_{3,32}=320.2$, p<0.0001; Treatment: $F_{1,32}=0.31$, p=0.58) and 14 days (d, $t_8=0.69$, p=0.51) after SCI. (**e**,**f**) MFI for CD45 on macrophages 1, 3, 7, and 21 days (e, Interaction: $F_{3,32}=0.51$, p=0.68; Time: $F_{3,32}=6.96$, p=0.001; Treatment: $F_{1,32}=0.02$, p=0.89) and 14 days (f, $t_8=0.19$, p=0.86) after SCI. (**g**,**h**) MFI for CD11b on macrophages 1, 3, 7, and 21 days (g, Interaction: $F_{3,32}=0.22$, p=0.88; Time: $F_{3,32}=24.51$, p<0.0001; Treatment: $F_{1,32}=0.13$, p=0.72) and 14 days (h, $t_8=0.27$, p=0.79) after SCI. (**i**,**j**) MFI for CD45 on granulocytes 1, 3, 7, and 21 days (i, Interaction: $F_{3,32}=0.19$, p=0.90; Time: $F_{3,32}=40.31$, p<0.0001; Treatment: $F_{1,32}=0.40$, p=0.53) and 14 days (j, $t_8=0.12$, p=0.91) after SCI. (**k**,**l**) MFI for CD11b on granulocytes 1, 3, 7, and 21 days (k, Interaction: $F_{3,32}=0.20$, p=0.90; Time: $F_{3,32}=34.03$, p<0.0001; Treatment: $F_{1,32}=0.88$, p=0.35) and 14 days (l, $t_8=1.08$, p=0.31) after SCI. (**m**) MFI for CD45 on T cells 1, 3, 7, and 21 days after SCI (Interaction: $F_{3,32}=1.25$, p=0.31; Time: $F_{3,32}=25.92$, p<0.0001; Treatment: $F_{1,32}=0.58$, p=0.45). (**n**) MFI for CD45 on $CD45^+CD11b^-$ lymphocytes 14 days after SCI (b, $t_8=1.41$, p=0.19). Two-way ANOVA and multiple unpaired t-test with n=5/group/time point. Data are presented as mean±SEM. *p<0.05.

Table S3. Changes in $CD45^+$ cell populations in the lesion area after SCI.

	Saline	XPro1595	Multiple t-test, p-value
Microglia (%)			
1d after SCI	32.15 ± 6.93	29.22 ± 2.37	t_8=0.40, p=0.70
3d after SCI	21.75 ± 1.76	20.41 ± 2.32	t_8=0.46, p=0.66
7d after SCI	36.47 ± 4.32	38.45 ± 3.81	t_8=0.34, p=0.74
14d after SCI	48.77 ± 8.72	68.03 ± 3.30	t_8=2.07, p=0.07
21d after SCI	49.82 ± 1.03	49.73 ± 4.06	t_8=0.02, p=0.98
Leukocytes (%)			
1d after SCI	50.56 ± 7.64	52.93 ± 1.54	t_8=0.30, p=0.77
3d after SCI	52.82 ± 2.37	52.72 ± 2.93	t_8=0.03, p=0.98
7d after SCI	38.30 ± 4.38	30.15 ± 1.86	t_8=1.72, p=0.12
14d after SCI	15.36 ± 2.71	7.664 ± 0.90	**t_8=2.70, p=0.03**
21d after SCI	20.03 ± 0.96	20.95 ± 2.26	t_8=0.37, p=0.72
Macrophages (%)			
1d after SCI	4.56 ± 0.66	4.36 ± 0.29	t_8=0.28, p=0.78
3d after SCI	4.43 ± 0.53	4.09 ± 0.40	t_8=0.51, p=0.62
7d after SCI	3.39 ± 0.26	2.31 ± 0.09	**t_8=3.89, p=0.005**
14d after SCI	2.80 ± 0.54	1.92 ± 0.28	t_8=1.46, p=0.18
21d after SCI	1.58 ± 0.14	1.79 ± 0.29	t_8=0.64, p=0.54
Granulocytes (%)			
1d after SCI	22.44 ± 3.90	23.49 ± 1.18	t_8=0.26, p=0.80
3d after SCI	17.50 ± 1.87	21.02 ±1.94	t_8=1.30, p=0.23
7d after SCI	21.08 ± 3.26	13.86 ± 3.22	t_8=1.58, p=0.15
14d after SCI	9.55 ± 1.53	3.83 ± 0.60	**t_8=3.89, p=0.008**
21d after SCI	9.85 ± 1.39	10.63 ± 1.90	t_8=0.33, p=0.75
T cells (%)			
1d after SCI	0.95 ±	1.02 ±	t_8=0.24, p=0.82
3d after SCI	1.34 ±	1.38 ±	t_8=0.18, p=0.86
7d after SCI	4.95 ±	6.56 ±	t_8=1.87, p=0.10
21d after SCI	5.66 ±	3.91 ±	**t_8=2.79, p=0.02**
$CD45^{high}CD11b^-$ cells (%)			
14d after SCI	8.60 ± 1.62	3.90 ± 0.71	**t_8=2.65, p=0.03**

*p<0.05, **p<0.01; multiple unpaired t-test, n=5/treatment group/time point. Data are presented as mean±SEM.

Table S4. Changes in CD45⁺ cell populations in the peri-lesion area after SCI.

	Saline	XPro1595	Multiple t-test, p-value
Microglia (%)			
1d after SCI	43.12 ± 5.67	44.35 ± 4.85	t_8=0.17, p=0.87
3d after SCI	55.78 ± 1.79	52.16 ± 5.02	t_8=0.68, p=0.52
7d after SCI	36.65 ± 5.17	45.89 ± 5.77	t_8=1.19, p=0.27
14d after SCI	53.98 ± 4.06	68.78 ± 3.23	**t_8=2.85, p=0.02**
21d after SCI	38.91 ± 6.51	52.46 ± 5.93	t_8=1.54, p=0.16
Leukocytes (%)			
1d after SCI	28.63 ± 1.85	31.28 ± 6.16	t_8=0.41, p=0.69
3d after SCI	22.51 ± 2.61	27.07 ± 5.18	t_8=0.79, p=0.45
7d after SCI	41.84 ± 4.99	31.13 ± 2.80	t_8=1.87, p=0.10
14d after SCI	14.02 ± 1.51	10.71 ± 2.13	t_8=1.27, p=0.24
21d after SCI	32.16 ± 3.87	23.22 ± 3.97	t_8=1.61, p=0.15
Macrophages (%)			
1d after SCI	3.58 ± 0.54	3.38 ± 0.26	t_8=0.33, p=0.75
3d after SCI	1.59 ± 0.39	1.98 ± 0.39	t_8=0.69, p=0.51
7d after SCI	4.26 ± 0.73	2.35 ± 0.35	**t_8=2.37, p<0.05**
14d after SCI	1.87 ± 0.31	2.81 ± 0.56	t_8=1.48, p=0.18
21d after SCI	2.98 ± 0.47	1.92 ± 0.33	t_8=1.83, p=0.10
Granulocytes (%)			
1d after SCI	9.50 ± 1.36	13.02 ± 4.68	t_8=0.72, p=0.49
3d after SCI	10.25 ± 1.51	12.95 ± 3.49	t_8=0.71, p=0.50
7d after SCI	28.22 ± 3.62	21.65 ± 2.09	t_8=1.57, p=0.15
14d after SCI	9.02 ± 1.91	6.14 ± 1.47	t_8=1.20, p=0.27
21d after SCI	19.85 ± 3.25	14.29 ± 3.12	t_8=1.24, p=0.25
T cells (%)			
1d after SCI	0.96 ± 0.24	1.05 ± 0.18	t_8=0.30, p=0.77
3d after SCI	0.95 ± 0.22	0.55 ± 0.06	t_8=1.71, p=0.13
7d after SCI	4.75 ± 0.70	6.20 ± 1.88	t_8=0.72, p=0.49
21d after SCI	3.27 ± 0.54	3.72 ± 0.51	t_8=0.61, p=0.56
$CD45^{high}CD11b^{-}$ cells (%)			
14d after SCI	10.61 ± 2.09	4.96 ± 1.01	**t_8=2.44, p=0.04**

*p<0.05, **p<0.01; multiple unpaired t-test, n=5/treatment group/time point. Data are presented as mean±SEM.
